# Ohio State Dental Society—Discussions

**Published:** 1871-01

**Authors:** 


### OHIO STATE DENTAL SOCIETY—DISCUSSIONS.

Dr. L. Buffett, of Cleveland, offered the following resolution:

*Resolved*, That no one shall be eligible to membership in this Society unless he have a diploma from some reputable Dental College, or a certificate from the State Board of Examiners.

Dr. W. M. Herriott thought the resolution unnecessary. A committee, composed of Drs. Decamp, Rehwinkle, and Smith, had been appointed by the Society, and after having the matter under consideration for a year had not recommended any change. He also thought the matter need not be brought up at this early stage of the meeting, to waste time in discussion. If at the close it was thought best, such a resolution could be passed to govern action next year.

Dr. Buffett thought the report of the committee referred to had no bearing on the case.

Dr. B. T. Spellman favored the resolution. Dr. Buffett, who offered it, had been one of the committee of last year. The action of that committee he thought, did not bind this body. Bodies bind committees, committees never bind bodies, The committee failed to make a report. It is competent for the Society to make the rules that the committee failed to make. It becomes imperatively necessary to have something of this kind. Here the old members who have been in the Association for years, are called upon every year to pay the expenses of the State Board, while the very men who should sustain it are not doing so, but are getting the credit all the same. The resolution he thought, ought to have been adopted before this time.

The limited time given to the Committee on Membership made it impossible for them to make such an examination as they should. They come up here to attend to other matters of great importance, and because of the little time the committee have, these candidates are admitted without much examination. It would be humiliating if, after they are admitted, they should fail when they go before the State Board. They should go before this Board first, and then the committee would not need to examine them on the same points.

Dr. Harroun expressed himself in favor of the resolution. If persons are admitted into this Society before it was known whether they were qualified or not, or before the State Board knows it, it seemed to him a lame kind of proceeding. It is no more than right that they should be previously examined by the State Board. They ought to feel that this is a duty they owe to themselves and to the people of the State.

Dr. Buffett being called to the chair, Dr. Rehwinkle said that the duties of the Committee on Membership was, to a great extent, confined to the qualifications of members, but they could go far beyond that. A man may be a dentist in Ohio or the United States, well qualified for his profession, yet may be utterly unfit to be a member of this Society. His private character may be such that none of us would tolerate him at our meetings; therefore the functions of the

Committee on Membership are different from those of the Board of Examiners, which merely has to do with a man's professional qualifications.

A strong reason why members who are not qualified should not be admitted to membership is this: if a man is received and remains a member in good standing until the expiration of his probation, which will be in 1872, and should then fail to pass the Board of Examiners and be prosecuted, it could be said to us, that two or three years ago we admitted him to membership in the Society, but now say he is not qualified. By admitting him to membership we acknowledge that he is our equal.

Dr. Herriott thought as the Society knew what his opinion was with regard to the law as it now exists, and of the proper status of a man who would practice dentistry, it was not necessary for him to make any explanations in regard to the qualifications that should be possessed by those entering the profession. But he did not believe it to be the object of the Society merely to have those who have a certificate from the State Board to meet together and exclude all others. He thought it should be their object to *educate* the profession, and to bring up those not so well qualified, to the point to which the best members aspired. They should have the sympathy and assistance of the older and better qualified members of the Society.

Dr. Watt was in favor of all the societies in the State, except the Ohio State Dental Society, doing exactly what Dr. Herriott recommended. The Mississippi Valley Dental Society, or the Central Society might take rising men and fit them to receive a diploma from the State, but the *State* Society stands in a different relation. That Society elects the State Board. "Now suppose," said he, "that in our infinite mercy we admit every young man that comes along whom we believe to be honest and upright, and that just before 1872, they begin to think it is about the time the screws are going to be turned upon them and they go to work and elect a merciful Board—and they would be just as sure to pick on him as Chairman of that Board, as could be." [Laughter.] By electing such a Board, all could pass it whether qualified for the profession or not. The State Society is responsible for the action of the Board; or in other words, it is responsible for the execution of that law. He believed it to be suicidal to receive into the Society those who have ceased to be lawful practitioners, or who fail to pass the State Board.

Dr. G. W. Keely, of Oxford, thought it not best to rush this matter through in too much haste. He liked the doctrine of his friend the Secretary, but still, he thought they would, perhaps, have to come to the point contemplated in the resolution. That all might have further time to consider the matter, he moved that the subject be postponed until 2 P. M., the next day. The motion was carried.

### "THOUGHTS ON RESPIRATION."

After Dr. Watt had read a paper* upon the above subject, Dr. J. Taft said there was one point upon which he would like to make an inquiry; that was the point at which the evolution of force takes place. He supposed it to be at the time and place that the change is made in the tissue and the change made in the blood, but he would like to know something further about the evolution of life forces, and how far it is modified by this mutation of material.

Dr. Watt in reply, said he supposed there never was a man passing through a beautiful country that went so far that he did not want to go farther; he then made some explanation of the principles contained in the paper. He did not believe, in this state of existence, the mind could be exercised without producing a physical change, or a chemical change in the tissue of the brain. Some day, perhaps, the chemist would be able to determine to some extent, the varied operations of

---

* See original department.

the mind by an analysis of the resulting materials. If a man has been engaged in vigorous thought, it can now be detected by the large amount of phosphates in his urine. The spirit working through the brain causes the change of force to take place, resulting in voluntary action. It was claimed by some that thought is the result of organization, but he believed that the reverse was true, and that organization is the result of thought. Give a spirit matter, and that spirit will make itself a body if it needs one. That is the tendency—if that spirit has sufficient spiritual force to control this matter. The Great Spirit of all had such force of spirit and spoke bodies into existence.

As an illustration of the superiority of spirit over matter, he related the following: A woman had been in ill health and for years was confined to her bed, suffering a great deal. She had become very feeble, and breathed with difficulty. The house took fire and the children were about to be burned. The fire had originated in a closet under the stairway, and the children were up stairs. That mother, however, did not allow her children to burn to death because the stairway was on fire. She got up from her bed, took hold of the bureau, and calling her husband, assisted in carrying it out and placing it beneath a window, and requesting her husband to get upon it, she procured a chair which he placed upon it and getting upon that he was enabled to rescue the children. In this case there was not much of the oxygen of reserve spoken of in the paper, but the aroused will controlled and used the needed supply.

He remarked that there was nothing peculiar in the oxygen in the blood. We have the two conditions, active and passive, the same that exists everywhere, and this element behaves in precisely the same manner in the body as it does out of it.

Dr. Spellman said that he understood the paper to assert that the oxygen that enters into the combinations that compose the animal tissue is derived entirely from the food. He inquired if the oxygen of the atmosphere did not contribute to the production of animal tissue.

Dr. Watt replied that animal life could not be sustained without food containing these four elements, oxygen, nitrogen, carbon, and hydrogen. These four elements are all combined in the food. This is the organic compound. Though the air contains three of these elements, the oxygen which reaches the blood by respiration is not combined with these other elements, but is oxygen free, and instead of uniting with the other oxygen, which is a part of the organic compound, its tendency would be to dispute with it the possession of these other elements. In other words the oxygen that is taken in with the food and is assimilated, passes through in combination with the other elements, and results in the formation of an organic compound; but the oxygen received by respiration is oxygen free, whether it is active or passive, and being free it is ready to combine with anything that is oxydizable, and especially with the protoxyd of iron. Then, the remainder of the oxygen not necessary to sesqui-oxydize that iron, passes on, ready whenever rendered active, to combine with oxydizable matter.

Dr. Spellman asked if it is a physiological fact that there is an excess of oxygen in the blood vessels, going along there hoping it may be taken up by some mishap that may occur?

Dr. Watt replied: No, not hoping for anything of the kind. But it is going along there ready to obey the will. It is a physiological fact, only demonstrated within the last few years by a number of experiments, that oxygen is taken in, in greater quantity in sleep, than when we are working actively, and that we give out a great deal more oxygen than we take in during the day; the best we can do when awake is to take in enough oxygen to carry on the involuntary operations of the system, so it is necessary to sleep in order to lay in the supply required. We have been trying to teach that doctrine theoretically for a dozen years, but it has only been practically demonstrated recently.

### EVENING SESSION.

### "DISEASES OF THE ANTRUM."

Dr. Buffett said that most of the diseases referred to seldom fall into the hands of dentists for treatment. A majority of the cases coming under their care is an inflamed condition of this membrane, which arises from disease or laceration of the periosteum. The antrum is subject to all those conditions which affect cavities lined with the mucous membrane. In relation to the treatment, he would say that if it was an inflamed condition he would remove the cause; sometimes this may require the removal of the tooth, sometimes saving the tooth.

Dr. Herriott agreed with Dr. Buffett that few cases, comparatively, of this disease comes under our care, yet when a case does come, we should be able to treat it properly. He mentioned a couple of cases he had treated for this disease. In the first case where the first bi-cuspid tooth had penetrated the cavity of the antrum, he endeavored first to treat the case, without removing the tooth, but finally removed it and treated with chlorid of zinc as strong as the patient could bear it; afterwards only Port wine was used, and then simply syringed with water. He would never treat a case of diseased antrum unless the patient could come to his office and allow him to use the remedies he desired. He had treated the case referred to about four weeks when the patient was dismissed. This had been some eighteen months or two years since and the cure was permanent.

The other case treated was caused by one of those fellows who travel through the country with a carpet-sack. He had endeavored to extract the outside roots of the second molar tooth, and instead of forcing the forceps alongside, had forced the root up into the cavity where it remained a year and a half. The patient had come to him and finding this piece of a tooth he removed it. The roots were joined together and in endeavoring to remove them he found considerable difficulty, as the orifice was not sufficiently large. The patient not being willing to take chloroform at his office, he had to go some distance in the country to perform the operation. He treated the case a little differently from the other, using carbolic acid in the place of chloride of zinc, and afterwards Port wine, and finally used nothing but water. It had been about a year since he treated the case, and it was apparently well.

Dr. Butler referred to a case spoken of by Dr. Jennings. He considered it somewhat complicated, and would term it maxillary abscess connected with diseased antrum. It had been over a year's standing before it came under observation. The general condition of the patient was bad; his vital power low and he was thin in flesh. The case had come under Dr. Jenning's notice, and he by request, had examined it. He understood that the case had under Dr. Jenning's treatment culminated in a cure.

Dr. Herriott inquired what the treatment was.

Dr. Jennings by request, stated that the treatment consisted simply in washing it with a weak solution of sulphate of zinc every day for two weeks, after that about once a week, and then a very weak solution of Port wine.

Dr. Watt thought it better to get at *general principles* than to cite individual cases. The latter was too much like going by a family receipt book. The treatment which was efficient in one case might do no good in another. If general principles could be arrived at and classified, they might have something to guide them. He extended his remarks to considerable length in suggesting some of the ways in which this generalization and classification might be accomplished.

Dr. J. Taft said there are a great variety of affections of this cavity. Taking into account the influences that would operate upon them, and tend to modify them, there would necessarily be a very great variety of affections of this kind. Even if all systems were alike, and the physiological and pathological conditions the same, still there would be considerable room for diversity; but since there is a very great variety of physiological conditions, and perhaps a still greater variety of pathological conditions, he was led to the conclusion that there was probably as great a variety of these affections as could be found anywhere else in the body. There may be simply an inflamed condition of this cavity manifesting itself in the various forms referred to, in which the producing cause may have been entirely local, and this may run along through different stages to that of a chronic condition, and may maintain that condition for an indefinite length of time; then again it may be brought under the influence of some further disturbing causes and go on to some more aggravated form of disease, and may take on the most violent or malignant character of disease of which human tissue is susceptible, and all character of grades between these extremes may exist either from local causes alone, or it may be, influenced greatly by systematic conditions—unfavorable conditions in the system. All these conditions and variations are so fully explained in medical works that they need not be discussed at our meetings, but must be made a matter of special study.

They should understand, that if the difficulty is an inflammation of the mucous membrane of the mouth, it takes on the nature of the same disease in mucous membranes elsewhere.

In other cases there may be a hypertrophied condition of the parts, so that they will be many times their normal thickness, and will require a different treatment from a mere chronic condition. In all such cases the dentist should be able to know the conditions, the tendencies, and the influences that will probably be operative in the case. Every dentist ought to understand the character of tumors on the gums or alveolus and know enough to ascertain whether they are benign or malignant, and, when necessary, be able to remove them as readily as the surgeon.

Dr. Decamp stated a case of a lady about forty-five years of age. Nearly all the teeth in the upper maxillary were decayed Upon examination he found a lump growing down inside of the cheek half as large as a hen's egg. It had been over a year growing. He took out he supposed, two table-spoonfuls of bloody matter, about the color of dark coagulated blood. The outer plate of the bone was entirely necrosed. All the treatment he gave was to syringe, in the first place, with diluted phenol. At the second sitting he removed all the necrosis of the bone he could, and continued the treatment with phenol diluted, which he laid on some cloth in the opening. After some time he extracted the remainder of the roots. The lady had been wearing an artificial plate for six or seven years.

Dr. Rehwinkle agreed with Dr. Taft that it was important for them to be able to diagnose properly in such cases as had been referred to. Even if dentists are not called upon to perform operations of that kind, it would be of incalculable benefit to them and their patients if they could promptly give an intelligent opinion about such cases, and to be able to determine whether diseases of that cavity are harmless, or whether they are likely to assume a malignant character. He then related a case where a lady some years since had him extract a tooth; in a couple of days she returned to the office with quite a violent hemorrhage from the cavity. He arrested the hemorrhage promptly with a moderate-sized ball of cotton, saturated with either chloride or sulphate of zinc, he did not remember which. He succeeded in driving it home, but being forced through the opening in the antrum he had a nice time in fishing it out, and as it came out by piece meal, he could not tell whether he had got it all out or not. He then rinsed out the cavity—it bleeding freely all the time—and succeeded by taking a larger amount of cotton, which he pressed into the bottom of the cavity, then taking more cotton and saturating it with persulphate of iron, and spreading it over and tying the jaws together to produce a pressure, he arrested the hemorrhage. In two or three days the patient returned, and he found that suppuration had begun. He noticed the case with a good deal of interest and watched continually for the cotton. It finally forced its way through the opening, clotted in a lump by the mucus, and that ended the trouble, the wound healing up rapidly.

In regard to the influence of malaria, touched upon by Dr. Taft, he said it seemed almost incredible. Such was its influence in his locality that a person could not have the toothache without its coming on at regular periods, and partaking of the nature of intermittent fever. A thorough study of the subject of such influences should be made by all, and especially by the younger members of the Society.

Dr. Taft remarked that the variation which is found in different localities would account, to a considerable extent, for the variation in cases so often presented by members of the profession. One will speak very positively about certain things in his experience, which another says can not be, because it never happened with him; but if all the facts were known, perhaps no two districts are affected alike in every respect. These influences all make their impress upon us. He remembered that in a certain district in Greene county there was a great amount of malaria. All the patients coming from that district gave a great deal of trouble, because of its influence, while others from other parts of the county gave no such trouble. This fact was so marked that he could not help noticing it.

Dr. T. A. Reamy, M. D., of Zanesville, being present was requested to address the meeting. He expressed himself as deeply interested in the discussions, and thanked the Society for the invitation to address them. He was glad to see the improvement made in the profession from year to year, and rejoiced in their advancement and prosperity.

Dentists would have to understand this subject of malaria, and the physiology of the entire system. They had trenched further and further upon the medical profession, and before the close of the nineteenth century a doctor of dentistry would have to be an excellent therapeutist, and must be a well educated man, or he would not be considered a good dentist.

He concluded with a few remarks upon the subject of malaria, which they had been discussing. This subject had ever been a mixed problem. It was not confined to low or damp localities alone, but was often prevalent upon lofty hills, while the inhabitants of lower districts and those living near water courses would be exempt. Philosophers and physicians had theorized upon it from time immemorial; yet it was an unsolved problem still. That it had a wonderful power all must admit, but it was like the power of the devil, its *influences* only could be seen as it went about continually seeking whom it might devour.

Dr. Watt was gratified to hear a prominent physician acknowledge the progress of the dental profession, and pleased to hear him express the belief that before the close of the present century, any one who would be a reputable dentist must be a man well educated in general medical science; he simply desired to make the doctor more glad by stating a fact that many did not know; that is, that there is but one college in Cincinnati that required two courses in practical anatomy in order to receive a degree, and that one is the Ohio College of Dental Surgery.

